# Adhesion to and biofilm formation on IB3-1 bronchial cells by *Stenotrophomonas maltophilia *isolates from cystic fibrosis patients

**DOI:** 10.1186/1471-2180-10-102

**Published:** 2010-04-07

**Authors:** Arianna Pompilio, Valentina Crocetta, Pamela Confalone, Mauro Nicoletti, Andrea Petrucca, Simone Guarnieri, Ersilia Fiscarelli, Vincenzo Savini, Raffaele Piccolomini, Giovanni Di Bonaventura

**Affiliations:** 1 Department of Biomedical Sciences, "G. D'Annunzio" University of Chieti-Pescara, Abruzzo, Italy; 2 Center of Excellence for Aging, "G. D'Annunzio" University Foundation of Chieti-Pescara, Abruzzo, Italy; 3 Laboratory of Microbiology, "Sant'Andrea" Hospital, Rome, Italy; 4 Department of Basic and Applied Medical Sciences, "G. D'Annunzio" University of Chieti-Pescara, Abruzzo, Italy; 5 Clinical Microbiology Laboratory, Pediatric Hospital "Bambino Gesù", Rome, Italy; 6 Clinical Microbiology and Virology, Department of Transfusion Medicine, "Spirito Santo" Hospital, Pescara, Italy

## Abstract

**Background:**

*Stenotrophomonas maltophilia *has recently gained considerable attention as an important emerging pathogen in cystic fibrosis (CF) patients. However, the role of this microorganism in the pathophysiology of CF lung disease remains largely unexplored. In the present study for the first time we assessed the ability of *S. maltophilia *CF isolates to adhere to and form biofilm in experimental infection experiments using the CF-derived bronchial epithelial IB3-1cell line. The role of flagella on the adhesiveness of *S. maltophilia *to IB3-1 cell monolayers was also assessed by using *fliI *mutant derivative strains.

**Results:**

All *S. maltophilia *CF isolates tested in the present study were able, although at different levels, to adhere to and form biofilm on IB3-1 cell monolayers. Scanning electron and confocal microscopy revealed *S. maltophilia *structures typical of biofilm formation on bronchial IB3-1 cells. The loss of flagella significantly (P < 0.001) decreased bacterial adhesiveness, if compared to that of their parental flagellated strains. *S. maltophilia *CF isolates were also able to invade IB3-1 cells, albeit at a very low level (internalization rate ranged from 0.01 to 4.94%). Pre-exposure of IB3-1 cells to *P. aeruginosa *PAO1 significantly increased *S. maltophilia *adhesiveness. Further, the presence of *S. maltophilia *negatively influenced *P. aeruginosa *PAO1 adhesiveness.

**Conclusions:**

The main contribution of the present study is the finding that *S. maltophilia *is able to form biofilm on and invade CF-derived IB3-1 bronchial epithelial cells, thus posing a rationale for the persistence and the systemic spread of this opportunistic pathogen in CF patients. Experiments using *in vivo *models which more closely mimic CF pulmonary tissues will certainly be needed to validate the relevance of our results.

## Background

Cystic fibrosis (CF) is a common inherited genetic disorder, caused by a mutation in the gene encoding the cystic fibrosis transmembrane conductance regulator (CFTR) protein [1] which is expressed in many different cells. In the lung, the derived chloride transport defect leads to altered airway physiology including impairment of mucociliary clearance, production of plugs of thick mucus and impaired innate immunity [2,3]. These defects predispose the CF patient to microbial colonization and thus, to infections that tend to become chronic. The likelihood of contracting chronic infections increases with age and *Pseudomonas aeruginosa *becomes the dominant infecting microorganism, with a colonization percentage varying from 42 to 100% [4].

Recently, *Stenotrophomonas maltophilia *has gained considerable attention as an important emerging nosocomial pathogen able to cause infections in debilitated and immunocompromised patients, as well as in CF patients [5,6]. Colonization of the pulmonary tissues occurs in approximately one third of CF patients, nevertheless, there is controversy as whether *S. maltophilia *colonization leads to a poorer clinical outcome or morbidity [7-9]. Persistent colonization by *P. aeruginosa *and the attendant damage of the epithelial mucosa by released pseudomonal exoproducts may increase the probability that *S. maltophilia *will colonize the respiratory tract of CF patients and significantly contribute to the progressive deterioration of their pulmonary functions [10,11]. However, the mechanism of pathogenicity enabling *S. maltophilia *to establish infection and chronic colonization of the respiratory tract of CF patients remains largely unexplored.

Biofilm formation is increasingly recognized as an important bacterial virulence trait contributing to disease progression in CF and other diseases of the respiratory tract associated with chronic infections. Biofilm growth is believed to protect bacteria from natural immune defenses, as well as from the actions of several antibiotic compounds [12,13]. *P. aeruginosa *strains isolated from the sputum of CF patients display morphologic and physiologic characteristics suggestive of *in vivo *biofilm formation, including over a 1000-fold increase in antibiotic resistance and a significant ability in evading host defense factors [14-17].

*S. maltophilia *has been recently reported to be able to adhere to cultured epithelial respiratory cells, as well as to produce biofilm on a variety of abiotic surfaces [10,18,19]. In particular, *S. maltophilia *strains isolated from CF patients were shown to be able, although with striking differences, to adhere to and form biofilm on polystyrene [20]. Since information on the ability of *S. maltophilia *to grow as biofilm in CF airway tissues is scarce, in the study described in this paper we evaluated, by quantitative assays and microscopic analysis (scanning electron and confocal laser microscopy), the ability of CF *S. maltophilia *strains to adhere, invade and form biofilm on CF-derived IB3-1 bronchial epithelial cell monolayers. Moreover, the role of flagella in adhesiveness on IB3-1 epithelial cells was also evaluated by the construction of two independent *S. maltophiia fliI *deletion mutants that were used to infect cultured monolayers. Some of the results of the present study have been previously presented in the form of an abstract at the 18^th ^European Congress of Clinical Microbiology and Infectious Diseases [21].

## Results

### *S. maltophilia *is able to adhere to and form biofilm on IB3-1 cell monolayers

We used IB3-1 human bronchial CF-derived cells to investigate the ability of *S. maltophilia *to adhere to and form biofilm. Confluent IB3-1 cell monolayers were independently infected with the 12 CF-derived *S. maltophilia *strains chosen for this study (Table 1); both the adhesiveness and the ability to form biofilm were measured by determining the number (cfu) of bacteria 2 and 24 hours post-infection, respectively. Growth curves, obtained with bacteria grown in MH broth, showed no significant differences in the mean generation time between isolates (mean ± SD: 3.35 ± 0.39 hours).

**Table 1:** Microbiological features of *S. maltophilia *OBGTC strains (n = 12) used in this study.

Strain	**Patient age**^**a**^	Co-isolated with:	**Chronic lung infection isolate**^**b**^	Past *P. aeruginosa *infection
OBGTC5	13	Pa, Ca	-	+
OBGTC9	17	Sa	+	+
OBGTC10	13	only	+	-
OBGTC20	11	Pa	+	+
OBGTC26	11	only	-	-
OBGTC31	16	Pa, Sa	+	+
OBGTC37	3	only	-	NA
OBGTC38	9	Sa	-	+
OBGTC44	16	Pa	+	+
OBGTC49	5	NA	+	+
OBGTC50	10	NA	+	+
OBGTC52	25	only	+	+

Caption and Abbreviations:^a^Ages shown are in years at the time of strain isolation. ^b ^Chronic infection is defined as the presence of two or more positive cultures for *S. maltophilia *in a year. Pa: *P. aeruginosa*; Ca: *C. albicans*; Sa: *S. aureus*; NA, not available.

**Figure 1:**
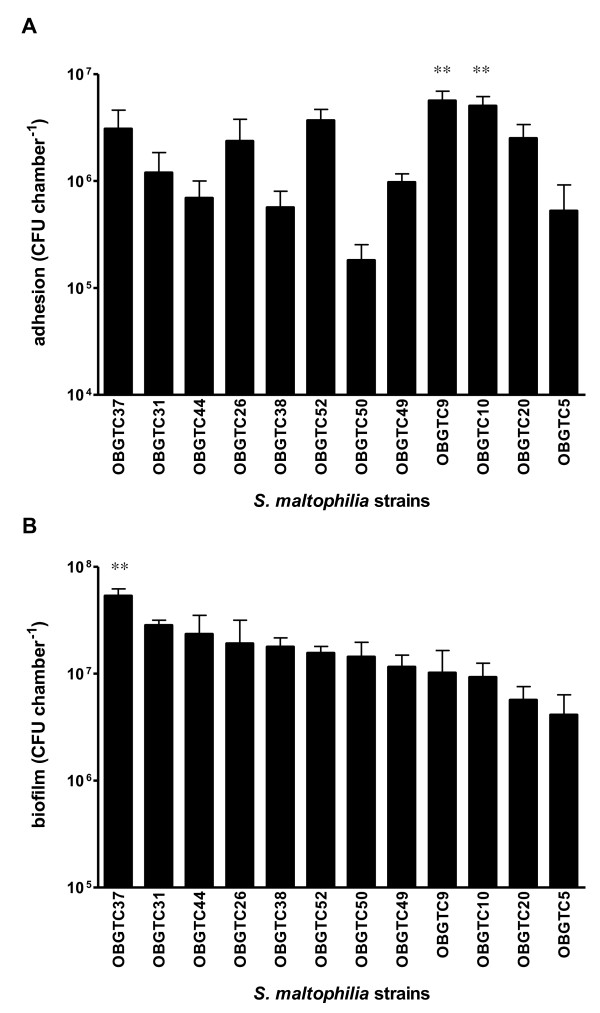
**Adhesion to and biofilm formation on IB3-1 cell monolayer of clinical isolates of *S. maltophilia *from CF patients**. **A**. Adhesion levels of *S. maltophilia *to IB3-1 cell monolayers. Strains OBGTC9 and OBGTC10 showed the highest level of adhesiveness, significantly higher than for the other strains (** P < 0.001; ANOVA-test followed by Newman-Keuls multiple comparison post-test). **B**. Biofilm formed by *S. maltophilia *on IB3-1 cell monolayers. Strain OBGTC37 formed the highest amount of biofilm, significantly higher (** P < 0.001; ANOVA-test followed by Newman-Keuls multiple comparison post-test) than other strains tested. Results are expressed as means + SDs.

All *S. maltophilia *strains tested were able to adhere to IB3-1 cells after 2 hours of incubation, with significantly different levels of adhesiveness among the strains (Figure 1A). *S. maltophilia *strains OBGTC9 and OBGTC10 showed the highest levels of adhesiveness (5.6 ± 1.2 × 10^6 ^and 5.0 ± 1.1 × 10^6 ^cfu chamber^-1^, respectively; P > 0.05), significantly higher if compared to that of the other strains (P < 0.001).

With regard to biofilm formation, as judged by the number of cfu recovered after 24 hours of incubation, *S. maltophilia *strain OBGTC37 produced the highest amount of biofilm (5.4 ± 0.8 × 10^7 ^cfu chamber^-1^) (Figure 1B), a value significantly higher if compared to the other strains tested (P < 0.001). No significant correlation was found between adhesiveness and the amount of biofilm formed (Pearson r, 0.158; P > 0.05).

**Figure 2:**
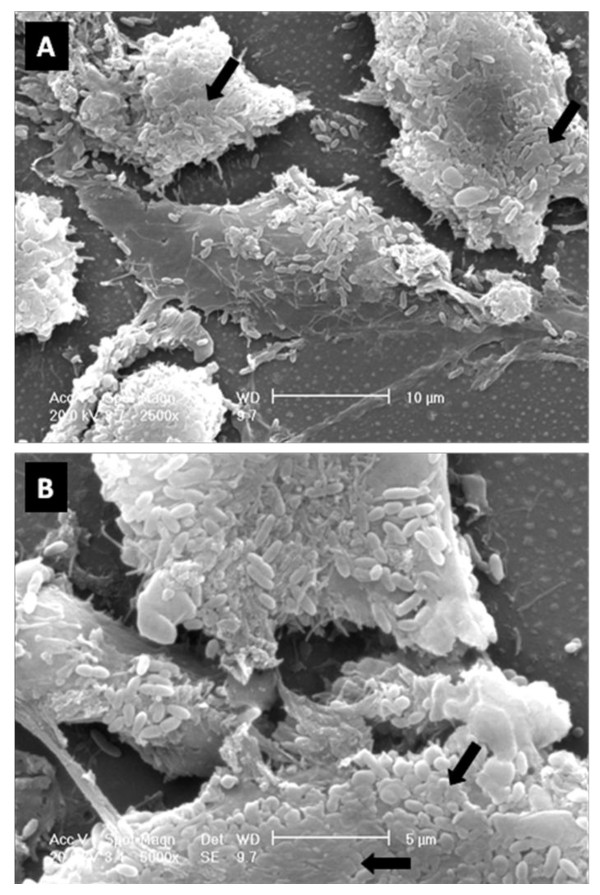
**SEM observation of 24 hours-biofilm formed byclinical isolate *S. maltophilia *OBGTC9 on IB3-1 cell monolayer**. Scanning electron micrographs showing cell cluster morphology (microcolony) strongly suggestive of biofilm formation. Bacterial cells lose their outlines for the presence of extracellular matrix (arrows). Magnification: ×2.500 (Figure 2A), ×5.000 (Figure 2B).

**Figure 3:**
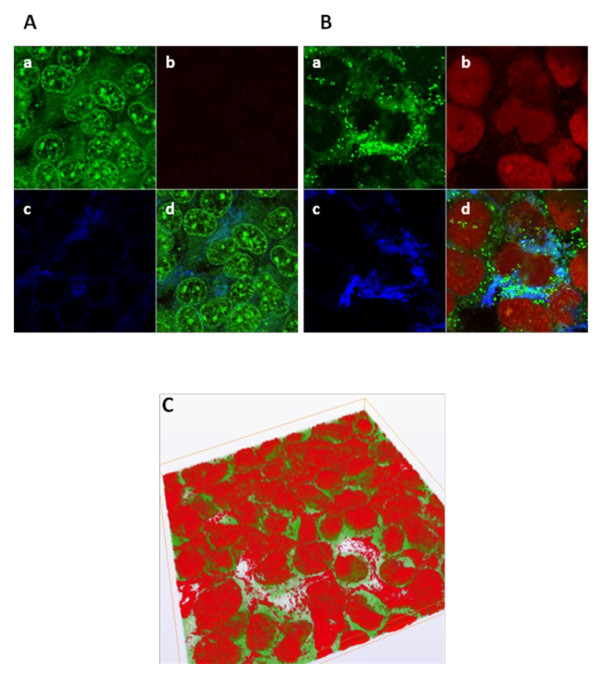
**CLSM observation of 24 hours-biofilm formed byclinical isolate *S. maltophilia *OBGTC9 on IB3-1 cell monolayer**. **A-B**. CLSM micrographs of not fixed specimens of unexposed (control; Figure 3A) and OBGTC9-exposed (Figure 3B) IB3-1 cell monolayer stained with Syto-9 (green fluorescence, indicating live cells), propidium iodide (red fluorescence, indcating dead cells), and Con-A (blue fluorescence, indicating extracellular matrix). Image capture was set for visualization of: (a) green fluorescence only; (b) red fluorescence only; (c) blue fluorescence only (3) or; (d) co-localization of all three fluorescence signals. Note the formation of a *S. maltophilia *microcolony embedded in matrix whose formation is significantly increased in infected vs control IB3-1 cell monolayers. **C**. CLSM examination of fixed IB3-1 monolayer exposed to *S. maltophilia *OBGTC9 for 24 hours: three-dimensional representation. Green fluorescence indicates autofluorescence of IB3-1 cytoplasm following exposure to fixation mixture; red fluorescence indicates binding of propidium iodide to nucleic acids of both IB3-1 and *S. maltophilia *cells. Note the microcolony organization of *S. maltophilia *on almost all IB3-1 cells. Magnification, ×100.

CLSM observation of IB3-1 cell monolayers infected for 2 or 24 hours with *S. maltophilia *showed no significant differences in cellular detachment with respect to control, thus confirming the integrity of exposed IB3-1 monolayers. Furthermore, after 24 hours of infection, both SEM and CLSM analysis revealed clusters of *S. maltophilia *cells scattered across almost all IB3-1 cells (Figures 2 and 3). CLSM analysis showed that microcolonies were embedded in extracellular matrix whose amount was significantly increased following infection (Figure 3B). These morphological observations are strongly suggestive of *S. maltophilia *biofilm formation on IB3-1 cells.

### Flagella are involved in *S. maltophilia *adhesion to IB3-1 cell monolayers

**Figure 4:**
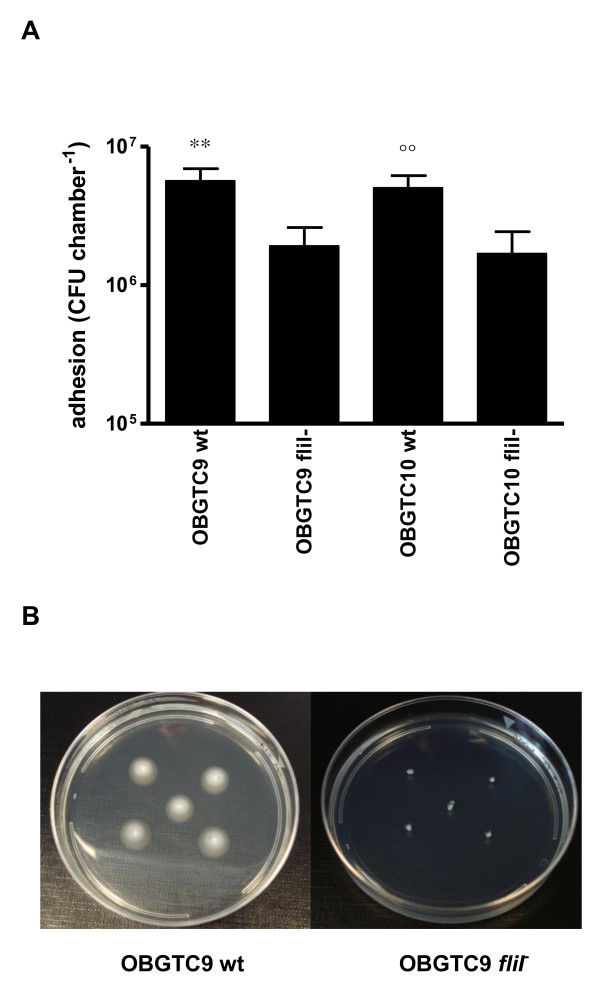
**Adhesion to IB3-1 cell monolayer by *S. maltophilia *OBGTC9 and OBGTC10 wild type strains, and relative *fliI*^- ^mutants**. **A**. The adhesiveness of OBGTC9 and OBGTC10 flagellar mutants fliI- was significantly lower than that of wild type strains (** P < 0.001 vs OBGTC9 *fliI*^-^; °° P < 0.001 vs OBGTC10 *fliI*^-^; ANOVA-test followed by Newman-Keuls multiple comparison post-test). Results are expressed as means + SDs. **B**. The inactivation of the *fliI *gene was confirmed by swimming motility assay: OBGTC9 wild type (left), and relative *fliI*^- ^mutant (right).

*S. maltophilia *has been shown to produce flagella implicated in the ability of bacteria to adhere to polystyrene [22]. To assess the role of flagella on the ability of *S. maltophilia *to adhere to IB3-1 cell monolayers, the adhesiveness of *fliI *mutant derivatives of *S. maltophilia *strains OBGTC9 and OBGTC10 was evaluated and compared to that of their parental wild-type strains by infecting IB3-1 cell monolayers, as described above. OBGTC9 and OBGTC10 were selected because they were the most adhesive in our group of strains (Figure 1A). As reported in Figure 4, the loss of flagella significantly (P < 0.001) decreased bacterial adhesiveness, if compared to that of their parental strains. We recovered 1.9 ± 0.6 × 10^6 ^cfu chamber^-1 ^from IB3-1 cells infected with the OBGTC9 *fliI *mutant vs. 5.6 ± 1.2 × 10^6 ^cfu chamber^-1 ^of the parental strain, and 1.7 ± 0.7 × 10^6 ^cfu chamber^-1 ^from cells infected with OBGTC10 *fliI *mutant vs. 5.0 ± 1.1 × 10^6 ^cfu chamber^-1 ^of the parental strain.

Contrary to wt strains, exposure of IB3-1 cells to OBGTC9 and -10 *fliI *mutant strains for 24 hours disrupted cell monolayer. Thus, results about biofilm formation by mutant strains are not available.

### *S. maltophilia *is able to adhere to and form biofilm on polystyrene

**Figure 5:**
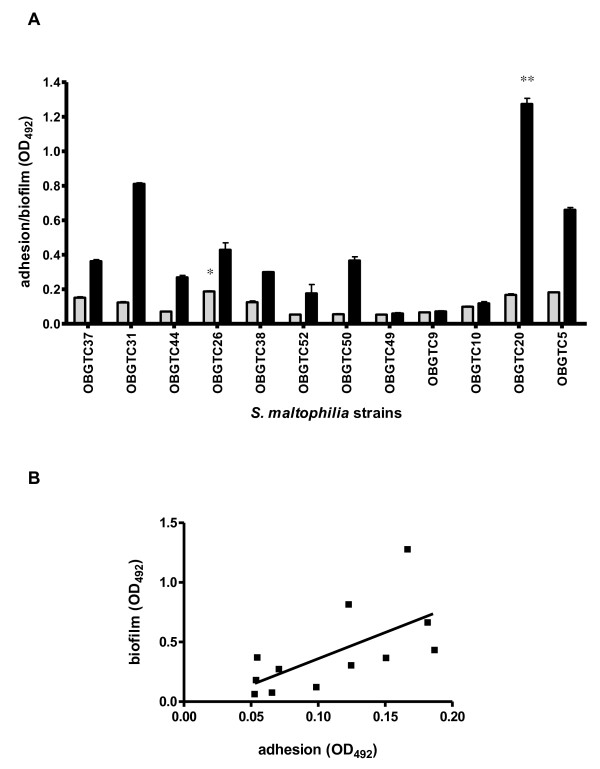
**Adhesion to and biofilm formation on polystyrene by 12 *S. maltophilia *isolates from CF patients**. **A**. Adhesion (grey bars) and biofilm (black bars) levels were assessed by crystal violet colorimetric technique and expressed as optical density read at 492 nm (OD_492_). OBGTC26 strain adhesiveness was significantly higher than OBGTC49, OBGTC50, and OBGTC52 strains (* P < 0.05; Kruskall-Wallis test followed by Dunn's multiple comparison post-test). Biofilm formed by OBGTC20 strain was significantly higher than that produced by OBGTC9 and OBGTC49 strains (** P < 0.01; Kruskall-Wallis test followed by Dunn's multiple comparison post-test). Results are expressed as means + SDs. **B**. Relationship between adhesion to and biofilm formation levels on polystyrene. A statistically significant positive correlation was found between adhesion and biofilm levels (Pearson r = 0.641; P < 0.05).

We then tested the ability of our *S. maltophilia *strains to adhere to and form biofilm on polystyrene plates. All twelve strains were found to adhere to and form biofilm on polystyrene plates, although with striking differences among strains (Figure 5A). Considering adhesiveness, the OD_492 _values (see Materials and Methods for details) ranged from 0.053 (strain OBGTC49) to 0.187 (strain OBGTC26). In particular, adhesiveness of strain OBGTC26 (0.187 ± 0.003) was significantly higher than that of strains OBGTC49, OBGTC50, and OBGTC52 (0.053 ± 0.002, 0.055 ± 0.003, and 0.054 ± 0.001, respectively; P < 0.05). Adhesiveness to polystyrene plates of the different strains did not correlate with their degree of adhesiveness to IB3-1 cells (Pearson r, -0.044; P > 0.05). With regard to biofilm formation, the OD_492 _values ranged from 0.060 (strain OBGTC49) to 1.274 (strain OBGTC20). In particular, biofilm formed by strain OBGTC20 (1.274 ± 0.032) was significantly higher than that produced by strains OBGTC9 and OBGTC49 (0.072 ± 0.003, and 0.060 ± 0.004, respectively; P < 0.01). Again, the ability to form biofilm on polystyrene plates of the twelve strains was not significantly correlated to their ability to form biofilm on IB3-1 cell monolayers (Pearson r, -0.127; P > 0.05). On the other hand, the results of the crystal violet staining showed a statistically significant positive correlation (Pearson r = 0.641; P < 0.05) between adhesiveness and ability to form biofilm (Figure 5B).

### *S. maltophilia *internalizes within IB3-1 cells at low levels

To ascertain whether our strains of *S. maltophilia *are able to enter IB3-1 cells, bacterial internalization was evaluated by a classical antibiotic exclusion assay. Due to high-level of gentamicin resistance, only 5 strains were tested for invasiveness. Gentamicin was highly effective on inhibiting the growth of the *S. maltophilia *strains (inhibition of growth ≥ 99.9%, data not shown) and was proved to be not toxic for IB3-1 cells even when they were exposed up to 1200 μg ml^-1^, as assessed by the XTT assay (data not shown).

The results of the invasion experiments indicated that all strains tested were able to invade IB3-1 cells, albeit at a very low level. Viable intracellular bacteria represented only a minor fraction of the total bacterial input used to infect cell monolayers.

Internalization rates (cfus released upon cell lysis, compared to cfus used to infect cell monolayers) were 0.54, 0.01, 4.94, 2.48, 0.03% for OBGTC9, OBGTC10, OBGTC37, OBGTC38, and OBGTC50, respectively. Internalization levels (expressed as number of internalized bacteria) were not significantly related to adhesion levels (expressed as number of adhered bacteria) (Pearson r: 0.044, P > 0.05).

### Swimming and twitching motilities are not involved in *S. maltophilia *adhesion to and biofilm formation on IB3-1 cells

The motility of our twelve *S. maltophilia *clinical isolates was assessed by swimming and twitching assays, as described in Materials and Methods. *S. maltophilia *strains exhibited a very broad range of motility (data not shown). Ten out of 12 (83.3%) strains showed swimming motility, ranging from 4 mm (strain OBGTC49) to 17 mm (strain OBGTC5). Strains OBGTC52 and OBGTC50 did not exhibit swimming motility. All strains were able to move by twitching, ranging from 3 mm (strain OBGTC49) to 15 mm (strain OBGTC37). Neither swimming nor twitching motility significantly correlated with adhesiveness to or biofilm formation on IB3-1 cells (data not shown). As expected, both OBGTC9 and OBGTC10 *fliI *deletion mutants failed to show swimming motility (Figure 4B).

### Pre-exposure to *P. aeruginosa *influences *S. maltophilia *adhesion to IB3-1 cell monolayers

**Figure 6:**
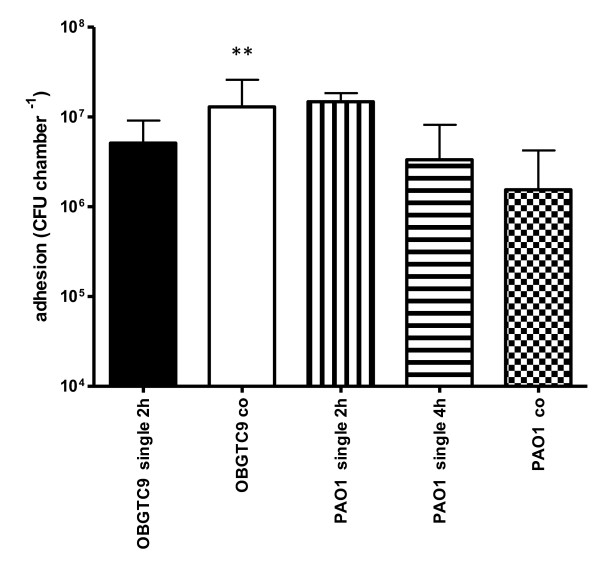
**IB3-1 cell monolayer co-infection assays**. IB3-1 cell monolayers were exposed first to *P. aeruginosa *PAO1 for 2 hours (PAO1 co), then for a further 2 hours to *S. maltophilia *OBGTC9 strain (OBGTC9 co). Control infections consisted of exposure for 2 hours to *S. maltophilia *OBGTC9 (OBGTC9 single 2 h) or *P. aeruginosa *PAO1 (PAO1 single 2 h). Results are expressed as means + SDs. Pre-exposure of IB3-1 cell monolayer to *P. aeruginosa *PAO1 significantly improved *S. maltophilia *OBGTC9 adhesiveness (** P < 0.01 vs OBGTC9 single 2 h; ANOVA-test followed by Newman-Keuls multiple comparison post-test). When IB3-1 cells were concomitantly infected, *S. maltophilia *OBGTC9 adhesiveness was significantly higher than that showed by *P. aeruginosa *PAO1 (** P < 0.001 vs PAO1 co; ANOVA-test followed by Newman-Keuls multiple comparison post-test).

It has previously been hypothesized that *S. maltophilia *colonization of pulmonary tissues of CF patients may be dependent on previous infections by strains of *P. aeruginosa *which, probably releasing not yet characterized exoproducts, induce damages of the pulmonary mucosa which may favor *S. maltophilia *colonization [12,13]. To get further insight on this phenomenon, we first infected IB3-1 cell monolayers with *P. aeruginosa *reference strain PAO1 for 2 hours at 37°C (MOI 1000), then rinsed three times with PBS, and finally incubated the cells with *S. maltophilia *strain OBGTC9 (MOI 1000) for further 2 hours. As control, we used monolayers separately infected with the two strains. The results obtained are summarized in Figure 6. When monolayers were separately infected, 2 hours-adhesiveness of *P. aeruginosa *PAO1 to IB3-1 cells was significantly higher than that of *S. maltophilia *OBGTC9 (1.5 ± 1.9 × 10^7 ^vs. 5.1 ± 3.9 × 10^6 ^cfu chamber^-1^, respectively; P < 0.01). However, when IB3-1 cell monolayers were first infected with *P. aeruginosa *PAO1 and then infected with OBGTC9, adhesiveness of *S. maltophilia *OBGTC9 was significantly improved, if compared to that of monolayers infected with only strain OBGTC9 (1.3 ± 1.3 × 10^7 ^vs. 5.1 ± 3.9 × 10^6 ^cfu chamber^-1^, respectively; P < 0.01). Moreover, when monolayers were concomitantly infected with both strains the adhesiveness of *S. maltophilia *OBGTC9 was significantly higher than that of *P. aeruginosa *PAO1 (1.3 ± 1.3 × 10^7 ^vs. 1.5 ± 2.7 × 10^6 ^cfu chamber^-1^, respectively; P < 0.001), even higher than that showed when monolayers were infected with *P. aeruginosa *PAO1 for 4 hours (3.3 ± 4.8 × 10^6 ^cfu chamber^-1^; P < 0.01), thus suggesting that the presence of *S. maltophilia *OBGTC9 negatively influences *P. aeruginosa *PAO1 adhesiveness.

## Discussion

Although recent clinical evidence highlights an increase in the frequency of isolation of *S. maltophilia *from respiratory tract of CF patients, the role of this microorganism in the pathophysiology of CF lung disease, as well as patient-to-patient spread, have not yet been clearly elucidated [5,7-9]. Moreover, the correlation between *S. maltophilia *persistent lung colonization and reduced pulmonary function first reported by Karpati et al [11], has not yet been confirmed by further studies [23-26]. On the other hand, the increased isolation of *S. maltophilia *from the sputa of CF patients has become a cause of concern in the CF community, as the organism is highly resistant to many of the antibiotics prescribed in CF management [27].

Because of its increasing clinical relevance, its high level of antibiotic-resistance, and the paucity of information on its specific role in the pathogenesis of CF lung infections, new information regarding the interactions between *S. maltophilia *and CF airway tissues are of paramount importance. To our knowledge, this is the first study which evaluated the ability of CF-derived *S. maltophilia *clinical isolates to adhere to and form biofilm in experimental infection experiments using the CF-derived bronchial epithelial IB3-1cell line.

Employing an *in vitro *static culture model, by using electron and confocal microscopy and determining the number (cfu) of attached bacteria at different time points post-infection, we showed that all the twelve studied CF-derived *S. maltophilia *isolates were able, although at different levels, to adhere and form biofilm when co-cultured with IB3-1 cell monolayers. Such results suggest that these characteristics might be highly conserved among *S. maltophilia *strains isolated from CF patients. Electron and confocal microscopy revealed *S. maltophilia *structures typical of biofilm formation on almost all bronchial IB3-1 cells. In particular, the overall cellular areas occupied by bacteria and their numbers are suggestive of the formation of microcolony, a finding reminiscent of the "flat" biofilm phenotype produced by *P. aeruginosa*, significantly different from the "mushroom-like" phenotype [28]. Electron microscopy photographs revealed that *S. maltophilia *adhered to IB3-1 cells loses its cell profile, probably due to the presence of extracellular matrix. In fact, CLSM examination showed microcolony embedded in extracellular matrix whose production was significantly increased following exposure to *S. maltophilia*.

The ability of *S. maltophilia *to form biofilm on IB3-1 cells may contribute to explain why *S. maltophilia *tends to produce persistent infections in chronic obstructive pulmonary disease despite intensive antibiotic treatment [29]. In this context, it is worth mentioning that some antibiotic-resistant *S. maltophilia *strains may persist in CF patients pulmonary tissue for up to 3 years, and that many patients are colonized at the same time with multiple strains of *S. maltophilia *[30].

Invasion of epithelial respiratory cells has been reported for CF-derived *S. maltophilia *clinical isolates [10,20]. We have recently reported that, with the exception of an environmental *S. maltophilia *isolate (strain LMG959) all the CF-derived strains assayed were able to invade A549 cells [20]. In the present study we evaluated the ability of twelve *S. maltophilia *CF isolates to invade IB3-1 cells, by classical invasion assays. The results obtained clearly indicated, for the first time, that *S. maltophilia *CF isolates were able to invade IB3-1 cells, albeit at a very low level (data not shown). Since strains presented a significant degree of heterogeneity in internalization efficiencies, it might be possible to hypothesize that *S. maltophilia *entry within IB3-1 cells may be strain-dependent. Together with the ability to form biofilm, the capability of *S. maltophilia *to enter IB3-1 might also explain the tendency of this microorganism to become persistent within CF pulmonary tissues, since within intracellular compartments it could find protection against host defenses and the reach of antibiotics. Moreover, internalization may likely influence the modulation of the inflammatory response of the infected host.

It has been reported that flagella could act as adhesins which play a role in bacterial binding to host mucosal surfaces as well as to abiotic surfaces [22,31]. To study the role of flagella in the adhesiveness of *S. maltophilia*, we generated two independent mutants presenting a deletion encompassing the *fliI *gene of *S. maltophilia *strains OBGTC9 and OBGTC10. *fliI *encodes a substrate-specific ATPase (FliI), an enzyme necessary to provide energy for the export of flagellar structural components in a wide range of bacterial species [32]. Swimming ability of the two mutant strains was almost completely abolished (Figure 4B). When co-cultured with IB3-1 cell monolayers, the two mutants showed a reduced capacity to adhere to IB3-1 cells, if compared to that of parental wild type strains (Figure 4A). Further, we showed that neither swimming nor twitching motilities were significantly associated to adhesion to or biofilm formation on IB3-1 cells. Thus, taken together, our results suggest that although flagella must play some role in *S. maltophilia *adhesiveness, regardless of their functionality, other structures must also be involved in this phenomenon, since the *fliI *mutation only attenuates, but not abolishes, the ability of *S. maltophilia *strains to adhere to IB3-1 cells.

We were not able to assess the role of flagella in *S. maltophilia *biofilm formation since exposure of IB3-1 monolayers to *fliI*^- ^mutant strains caused their disruption already after 6h-exposure. Further investigations are warranted to explain this apparent increased virulence following the loss of motility.

A number of methods have been developed for cultivation and quantification of biofilms [12], but no standardized protocol for assessment of biofilm formation has been established so far. Nevertheless, the microtiter plate method remains among the most frequently used assays for investigation of biofilm formation, and a number of modifications have been developed for the cultivation and quantification of bacterial biofilms [33]. Since *S. maltophilia *biofilm formation on abiotic surfaces is generally considered less relevant than biofilm formation on cultured epithelial cells or *in vivo*, in this study we assayed biofilm formation onto an abiotic surface and compared the results to the ability of our *S. maltophilia *strains to form biofilm on IB3-1 cells, as assessed by quantitative colony counts. In agreement with previously reported experiments [20,34], all the twelve *S. maltophilia *clinical isolates tested were able to form biofilm on both polystyrene and IB3-1 cultured epithelial cells. However, no correlation was found between quantitative biofilm formation on the abiotic surface and qualitative biofilm formation on cultured cell monolayers, thus suggesting that the microtiter plate assay may not be predictive of the ability of *S. maltophilia *to form biofilm *in vivo*. Several explanations may account for this discrepancy. The crystal violet assay is surely a less specific method, and it is likely that the dye might also stain negatively charged extracellular molecules, including cell surface molecules and polysaccharides present in the extracellular matrix in mature biofilms, thus influencing the outcome of the test. Further studies are certainly needed to clarify this point.

Recent studies from different laboratories have highlighted the importance of interspecies bacterial interactions in influencing bacterial virulence and response to antibiotic therapy, both in pulmonary infections of CF and non-CF patients [35,36]. In CF patients, there are several lines of evidence indicating the presence of a mosaic of diverse bacteria so that infections of CF pulmonary tissues are usually considered always polymicrobial [37]. Recently, Ryan et al. [38] have reported that the presence of *S. maltophilia *significantly influences, as through the synthesis of a diffusible signal factor, the architecture of *P. aeruginosa *biofilm formation and augments its susceptibility to polymyxins, recently re-introduced into clinical practice as anti-pseudomonal agents.

In general, *S. maltophilia *is very often co-isolated with *P. aeruginosa *from CF patients [6,25,39,40] and it has been hypothesized that infection by *P. aeruginosa *may enhance the chance of *S. maltophilia *to colonize CF pulmonary tissues [12,13]. If this is true, it is reasonable to hypothesize that *P. aeruginosa *might enhance the ability of *S. maltophilia *to adhere to and/or invade CF pulmonary tissues. In order to analyze this possibility we first infected IB3-1 cell monolayers with reference *P. aeruginosa *PAO1 strain, and then with *S. maltophilia *strain OBGTC9 (the most adhesive of our group of strains; Figure 1A). The results obtained showed that while *P. aeruginosa *PAO1 binds more efficiently to cell monolayers than does *S. maltophilia *OBGTC9, a previous exposition of IB3-1 cell monolayers to *P. aeruginosa *PAO1 significantly improves *S. maltophilia *adhesiveness; therefore, it suggests a synergistic relationship between these pathogens similarly to what reported by Saiman et al. [41] who found a synergistic relationship between *P. aeruginosa *and *P. cepacia*. Demonstrating this, most (9 out of 12, 75%) of *S. maltophilia*-positive CF patients considered in the present study was found to have been infected in the past with *P. aeruginosa *(Table 1).

## Conclusions

Although the pathogenic role of *S. maltophilia *in CF lung disease is unclear and subject to controversy, the results of the present study suggest that this microorganism should not be considered just a bystander in CF patients. In this respect, we have shown that : i) *S. maltophilia *is able to adhere to and invade CF-derived IB3-1 cultured bronchial epithelial cells; ii) the ability of *S. maltophilia *strains to form biofilm and to invade epithelial cells might account for the persistence and the systemic spread of this opportunistic pathogen in CF patients; iii) a previous infection by *P. aeruginosa *may have an impact on *S. maltophilia *colonization of CF pulmonary tissues.

Further experiments using in vivo models which more closely mimic CF pulmonary tissues are certainly needed to validate the relevance of our results. Furthermore, our model may be useful to study the different stages of the intricate relationships between *S. maltophilia *and the CF airway epithelium, if compared to the abiotic model method. This may help in the development of new strategies for preventive and/or therapeutic intervention against the factors that trigger CF airways colonization by *S. maltophilia*.

## Methods

### Bacterial strains and culture conditions

Twelve *S. maltophilia *strains, herein designated as OBGTC, were used in this study (Table 1). All strains were isolated from the respiratory secretions of CF patients admitted to CF Unit of Pediatric Hospital "Bambino Gesù" of Rome. The isolates were identified as *S. maltophilia *by conventional biochemical tests (API 20-NE System; BioMérieux, Marcy-L'Etoile, France). *P. aeruginosa *PAO1 was used as a reference strain in IB3-1 co-infection experiments with *S. maltophilia*. Strains were kept at -80°C and grown overnight at 37°C on Mueller-Hinton or Trypticase Soy broth or agar (Oxoid; Garbagnate Milanese, Italy).

IB3-1 cells (ATCC#CRL-2777) are transformed bronchial epithelial cells isolated from a pediatric CF patient who harbored the ΔF508/W1282X mutations within the CFTR gene. Cells were grown at 37°C in LHC-8 medium supplemented with 5% fetal bovine serum (FBS) (Gibco, Italy) in a 5% CO_2_atmosphere.

Bacteria used to infect cell monolayers were grown in Trypticase Soy broth overnight at 37°C, under agitation (90 rpm), washed twice in sterile phosphate buffered solution (PBS; Sigma-Aldrich, Milan, Italy) and resuspended in LHC-8 medium (BioSource; Camarillo, Ca) to an OD_550 _= 0.450, corresponding to about 5 × 10^8 ^cfu ml^-1^. The concentration (cfu ml^-1^) of each bacterial suspension used to infect cultured cells was always determined.

### Construction of *S. maltophilia *flagellar mutants (*fliI*^-^)

*S*. *maltophilia fliI *chromosomal knockout mutants of strains OBGTC9 and OBGTC10 were constructed by using the gene replacement vector pEX18Tc, as described by Hoang et al. [42]. Briefly, a 2509-bp fragment, encompassing the entire ORF of the *fliI *gene, was PCR-amplified from total DNA preparations of *S*. *maltophilia *K279a reference strain using primers *fliI*Fw [5'-GGGGGGATCCAAGTCCTTTCCGCCTTCGCT-3' (the bold sequence corresponds to a *Bam*HI restriction site)] and *fliI*Rv [GGGGGAAGCTTGACAACTTCAGCCGACCGCT-3' (the bold sequence indicates a *Hind*III restriction site)]. The PCR-amplified fragment was digested with *BamH*I/*Hind*III and then cloned into the multicloning site of plasmid pEX18Tc, digested with the same restriction enzymes, thus generating plasmid pEX18ap. Next, a 971-bp cloramphenicol resistance cassette was PCR amplified from plasmid pACYC184 using the primer pair *cat*Fw [5'GGGGGGCTGCAGGCACCTCAAAAACACCATCATACA-3' (the bold sequence corresponds to a *Pst*I restriction site)] and *cat*RV [5'-GGGGGGTCGACCAGGCGTTTAAGGGCACCAATA-3' (the bold sequence indicates a *Sal*I restriction site)]. To generate a 1321-bp deletion within the internal coding region of *fliI*, the amplified 971-bp fragment was *Pst*I/*Sal*I digested and then cloned into plasmid pEX18Tap which had previously been digested with the same enzymes, thus generating plasmid pPEX53ap. pPEX53ap was introduced into *E*. *coli *S17-1 and independently mobilized into *S*. *maltophilia *strains OBGTC9 and OBGTC10 via conjugation. Transconjugants were selected on LB agar supplemented with 20 μg ml^-1 ^of tetracycline, 10 μg ml^-1 ^of cloramphenicol and 10 μg ml^-1 ^of kanamicin. Emerging resistant colonies were streaked on LB agar supplemented with 10% (wt vol^-1^) sucrose and then incubated overnight at 37°C. On the following day, sucrose-resistant colonies were screened for cloramphenicol resistance by growing individual colonies in LB plates supplemented with cloramphenicol. The inactivation of the *fliI *gene in chloramphenicol resistant colonies was confirmed by PCR amplification, Southern blot hybridization (data not shown) and swimming motility assays.

### Adhesiveness and biofilm formation on IB3-1 cultured monolayers

The ability of the twelve *S. maltophilia *strains and of the two independent OBGTC9 and OBGTC10 *fliI *deletion mutants to adhere to and form biofilms on IB3-1 cell monolayers was assayedusing a static co-culture model system. Briefly, IB3-1 cells were cultured in 8-well polystyrene chambers (CultureSlides, BD Falcon, Milan, Italy) seeded with 3 × 10^5 ^cells chamber^-1^, and grown to confluence in LHC-8 complete medium at 37°C and 5% CO_2_. Before seeding, wells were coated with 0.01 mg ml^-1 ^human fibronectin (BD Falcon), 0.03 mg ml^-1 ^bovine type 1 collagen (BD Falcon), and 0.01 mg ml^-1 ^bovine serum albumin (Sigma-Aldrich). Monolayers were infected with approximately 2.5 × 10^8 ^cells of each *S. maltophilia *strain analyzed, suspended in LHC-8 medium to obtain a multiplicity of infection (MOI) of approximately 1000, relative to the number of cells originally seeded. After 2 (adhesion assay) or 24 hours (biofilm assay) of incubation at 37°C, infected monolayers were washed three times with PBS to remove non-adherent bacteria and treated with 0.25% trypsin/EDTA (Sigma-Aldrich) for 10 minutes. Cells were recovered and then vortexed for 3 minutes, serially diluted, and bacteria plated on MH agar to determine the number (cfu chamber^-1^) of bacteria which adhered to IB3-1 cells.

Epithelial-monolayer integrity was assessed at 2 and 24 hours post-infection by confocal laser scanning and phase-contrast microscopy.

### Bacterial internalization assays

As described above, confluent IB3-1 cell cultures were infected with *S. maltophilia *strains (MOI 1000). After 2 hours of incubation at 37°C, infected monolayers were extensively washed with sterile PBS, and further incubated for other 2 hours in LHC-8 medium supplemented with gentamicin sulphate (600 μg ml^-1^; Sigma-Aldrich) in order to kill extracellular bacteria. We had previously determined that, at this concentration, gentamicin inhibits *S. maltophilia *growth by 99.9% (data not shown). At the end of the experiments, infected monolayers were extensively washed in PBS, then lysed with a solution of 0.1% Triton X-100 (Sigma-Aldrich) in PBS for 10 minutes at room temperature to count internalized bacteria. Aliquots of cell lysates were serially diluted and plated to quantify viable intracellular bacteria (cfu chamber^-1^).

Evaluation of toxicity of gentamicin towards IB3-1 cells was assessed by an XTT-based colorimetric assay (Cell Proliferation Kit II; Roche, Milan, Italy). Briefly, 500 μl of a mixture of XTT (1 mg ml^-1^) supplemented with 1.25 mM N-methyl dibenzopyrazine methyl sulfate was added to the wells containing cells incubated for 2 hours in LHC-8 medium supplemented with different concentrations (150 to 1200 μg ml^-1^) of gentamicin. IB3-1 cells not treated with gentamicin were used as control. Absorbance of supernatants was then measured at 492 nm in an ELISA plate reader (SpectraMax; Applied BioSystem Italia, Monza, Italy), subtracting background absorbance at 650 nm.

### Adhesiveness and biofilm formation on a polystyrene abiotic surface

Five-hundred microliters aliquots of bacterial cultures containing approximately 5 × 10^8 ^cfu ml^-1 ^were disposed on independent void wells of a sterile 48-wells flat-bottom polystyrene tissue culture plate (Iwaki; Bibby Scientific Italia, Riozzo di Cerro al Lambro, Milan, Italy). Bacteria were incubated at 37°C for 2 (adhesion assay) or 24 hours (biofilm assay) in a closed, humidified plastic container. At the end of the experiment, the medium was discarded, and non-adherent bacteria were removed by three washes with sterile PBS. Quantification of bacterial adhesiveness and biofilm formation on polystyrene was assessed by a spectrophotometric method, as previously described by Christensen et al. [43], with minor modifications. Briefly, after washing, attached bacteria were fixed for 1 hour at 60°C and then stained with Hucker crystal violet solution for 5 minutes. After washing with water to remove the excess of stain, the plates were dried for 30 minutes at 37°C. The color produced by attached bacteria (indirect index of adhesiveness or biofilm formation) was measured spectrophotometrically at OD_492_. A low cut-off corresponding to 3 standard deviations (SDs) above the mean of control wells not seeded with bacteria was chosen [43].

### Co-infection assays

Co-infection assays were performed using *S. maltophilia *strain OBGTC9 and *P. aeruginosa *strain PAO1. Briefly, confluent IB3-1 cell monolayers were first infected for 2 hours at 37°C with *P. aeruginosa *PAO1 (MOI 1000). At that time, non-adherent bacteria were removed by three washes with PBS, and monolayers were then infected with *S. maltophilia *strain OBGTC9 (MOI 1000) and incubated for further 2 hours. At the end of the experiment infected IB3-1 cells were removed by a treatment with 0.25% trypsin/EDTA, vortexed, serially diluted and plated on MH agar to determine the number (cfu chamber^-1^) of the two bacteria bound to IB3-1 cells. *P. aeruginosa *PAO1 and *S. maltophilia *OBGTC10 colonies were easily differentiated on the basis of their colonial morphology. As controls we used IB3-1 cell monolayers infected separately with each of the two bacterial strains.

### Motility tests

Swimming motility assays were performed with single well-isolated colonies grown overnight on MH agar plates, according to a modification of the technique described by Rashid et al. [44]. Briefly, tryptone swim plates (1% tryptone, 0.5% NaCl, 0.3% agar; Oxoid) were inoculated with bacteria at the surface by using a sterile needle. Plates were incubated for 24 hours at 37°C. Motility was assessed by calculating the diameter (mm) of the circular turbid zone formed by bacterial cells migrating away from the point of inoculation at the agar surface.

### Scanning electron microscopy

Biofilm formation was assessed by scanning electron microscopy (SEM). Samples were air-dried, and fixed with a solution of 2.5% glutaraldehyde in 0.1 M sodium cacodylate buffer for 90 minutes. After washing with buffer, samples were post-fixed in osmium tetroxide and then dehydrated in a series of aqueous ethanol solutions (30 to 70%). Specimens were mounted on aluminum stubs with conductive carbon cement, allowed to dry for 3 hours, and coated with 15-nm Au film with an agar automatic sputter coater. After processing, samples were observed with a Philips XL30CP scanning electron microscope in the high-vacuum mode at 15 kV.

### Confocal laser scanning microscopy (CLSM)

CLSM was carried out on fresh and formaldehyde-paraformaldehyde fixed samples. Briefly, infected IB3-1 cell monolayers, prepared as stated above, were stained with Live/Dead BacLight kit (Molecular Probes Inc.) and Concanavalin A (Alexa Fluor 647 coniugate; Molecular Probes Inc.). IB3-1 monolayer not exposed to *S. maltophilia *was used as control. CLSM analysis was performed with an LSM 510 META laser scanning microscope attached to an Axioplan II microscope (Zeiss). Three-dimensional reconstructions of imaged samples were obtained by Amira 3.1.1 (Mercury Computer Systems; Chelmsford, MA) software. Images were captured and processed for display using Adobe Photoshop (Adobe Systems Inc.) software.

### Statistical analysis

All experiments were performed in triplicate and repeated on two different occasions. Results were expressed as means ± SDs. Analyses of statistical significance were performed by ANOVA-test followed by Newman-Keuls multiple comparison post-test (adhesiveness and biofilm formation on IB3-1 cells, adhesiveness of *fliI *mutants, internalization within IB3-1 cell monolayers and co-infection experiments) or Kruskall-Wallis + Dunn's multiple comparison post-test (adhesiveness and biofilm formation on polystyrene). Interdependency between variables was evaluated by Pearson's linear correlation coefficient. P values < 0.05 were considered as statistically significant.

## Authors' contributions

APo, and PC performed the adhesion and biofilm formation assays on both polystyrene and IB3-1 cell monolayer. APo also carried out bacterial internalization assays, co-infection assays, motility tests, statistical analyses, and drafted the manuscript. VC took care of the additional experiments required during manuscript revision. MN and APe performed the construction of flagellar mutants. MN also participated in the revision of the manuscript. SG carried out microscopic analyses. EF and VS contributed by giving a medical point of view to the discussion of the results. EF also collected clinical strains used in the present work. RP, and GDB were involved in the design and coordination of the study, contributed to the revision of the manuscript, and gave their final approval of the version to be published. All authors read and approved the final version.
